# Antimicrobial peptides for bone tissue engineering: Diversity, effects and applications

**DOI:** 10.3389/fbioe.2022.1030162

**Published:** 2022-10-06

**Authors:** Zhuowen Hao, Renxin Chen, Chen Chai, Yi Wang, Tianhong Chen, Hanke Li, Yingkun Hu, Qinyu Feng, Jingfeng Li

**Affiliations:** ^1^ Department of Orthopedics, Zhongnan Hospital of Wuhan University, Wuhan, China; ^2^ Emergency Center, Hubei Clinical Research Center for Emergency and Resuscitation, Zhongnan Hospital of Wuhan University, Wuhan, China

**Keywords:** antimicrobial peptides, bone regeneration, topical applications, delivery system, gene therapy

## Abstract

Bone tissue engineering has been becoming a promising strategy for surgical bone repair, but the risk of infection during trauma repair remains a problematic health concern worldwide, especially for fracture and infection-caused bone defects. Conventional antibiotics fail to effectively prevent or treat bone infections during bone defect repair because of drug-resistance and recurrence, so novel antibacterial agents with limited resistance are highly needed for bone tissue engineering. Antimicrobial peptides (AMPs) characterized by cationic, hydrophobic and amphipathic properties show great promise to be used as next-generation antibiotics which rarely induce resistance and show potent antibacterial efficacy. In this review, four common structures of AMPs (helix-based, sheet-based, coil-based and composite) and related modifications are presented to identify AMPs and design novel analogs. Then, potential effects of AMPs for bone infection during bone repair are explored, including bactericidal activity, anti-biofilm, immunomodulation and regenerative properties. Moreover, we present distinctive applications of AMPs for topical bone repair, which can be either used by delivery system (surface immobilization, nanoparticles and hydrogels) or used in gene therapy. Finally, future prospects and ongoing challenges are discussed.

## 1 Introduction

Bone shows self-healing properties after trauma, but the repair of critical bone defects typically needs surgical therapies such as autogenous bone grafts, allogenous bone grafts and exogenous bone grafts (Zhai et al., 2020). In recent decades, bone tissue engineering shows great promise to become emerging strategy for surgical bone repair, which is mainly composed of four pillars including biomaterial scaffolds, bioactive factors, repair stem cells and biophysical stimuli (Hao et al., 2021). While great advances have been made in bone tissue engineering, the risk of infection during trauma repair remains a problematic health concern worldwide, especially for fracture and infection caused bone defects. It has been reported that the incidence of fracture-associated bone infections varies from 1.8% to 27% determined by the grade/type of fracture and fracture site (Birt et al., 2017). And bone defects caused by infections are generally correlated to pathogen recurrence. Once infection occurs during bone repair, pathogens may disturb bone regeneration and bone remodeling, resulting in further bone loss and causing enormous economic burden to the patient and society.

Bone infections may be mainly caused by three different mechanisms including contiguous contamination, hematogenous spread, and vascular or neurologic insufficiency (Birt et al., 2017). And common pathogens for bone infections are staphylococci such as *Staphylococcus aureus (S. aureus)* and streptococci. Other microbes such as anaerobes and Gram-negative anaerobic bacilli may also cause bone infections (Chen Y et al., 2021). Current antimicrobial strategies for bone defect infections are mainly based on systematic or topical administration of antibiotics. But antibiotic treatments are currently corrected with antimicrobial resistance because of improper over-reliance and over-prescription, which may cause infection recurrent, prolonged hospitalization and increased medical costs (Browne et al., 2020). Therefore, new generation of antimicrobial agents with limited resistance nature are highly needed in clinical practice.

Antimicrobial peptides (AMPs), also known as cationic host defense peptides (CHDPs), are emerging agents for infection treatments because of broad-spectrum antibacterial properties, controllable biocompatibility, diverse structures and categories. Compared with traditional antibiotics, AMPs rarely induce antimicrobial resistance by virtue of multiple antibacterial modes of action and their attacks to low-affinity targets such as bacterial membranes rather than a highly specific molecule (Zhang Q et al., 2021). Some AMPs, besides, also show immunomodulation capacities and regenerative effects such as cell proliferation and migration as well as angiogenesis. Considering that systemic application of AMPs is impeded by several shortcomings such as easily degradation in blood and rapid withdrawal by liver and kidney, topical administration of AMPs is highly recommended for trauma and infection repair.

**FIGURE 1:**
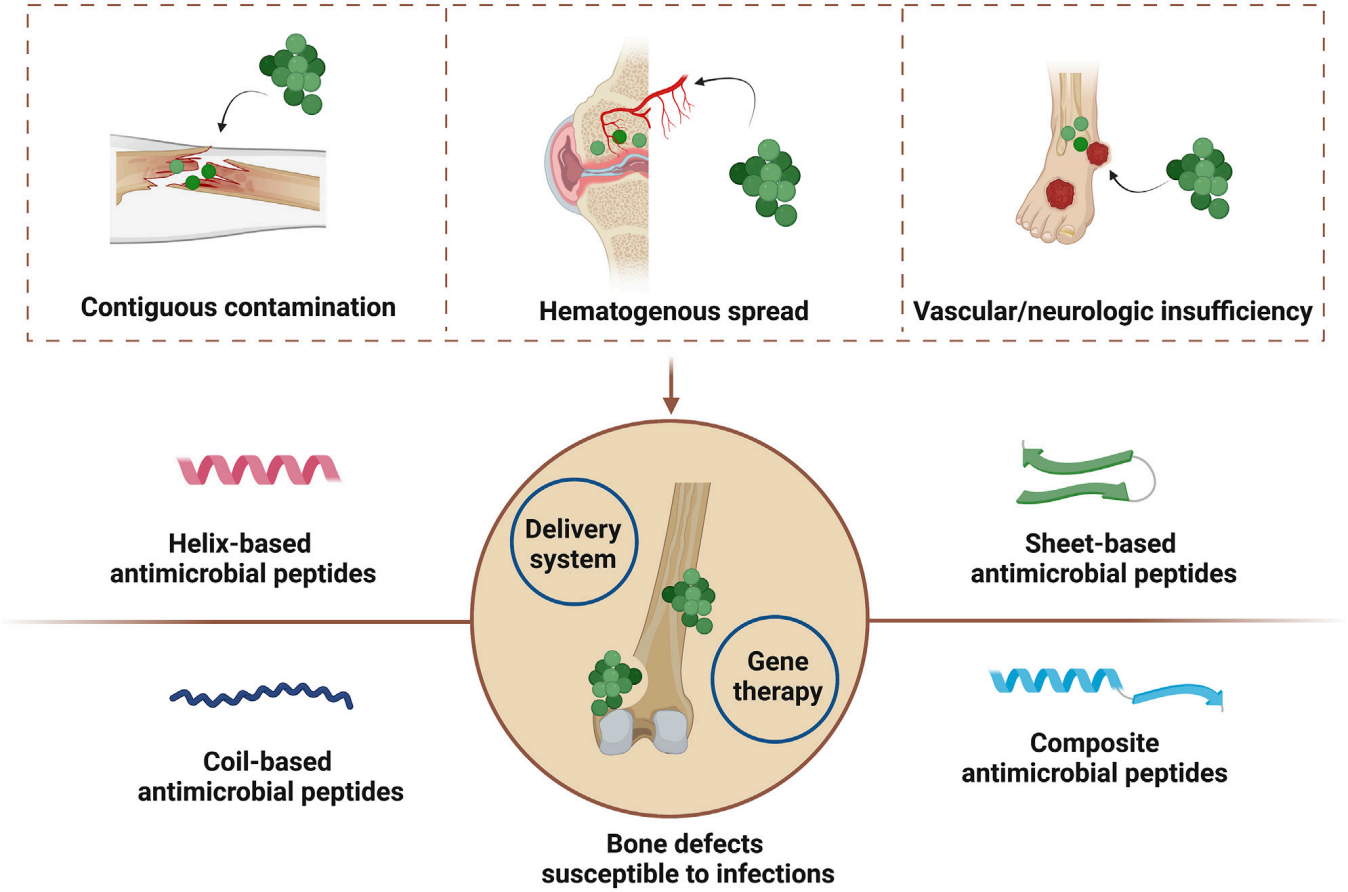
Mechanisms of bone infections and antimicrobial peptide-based strategies for the healing of bone defects susceptible to infections. Created with BioRender.com.

Currently, AMPs have been locally used for soft tissue repair such as diabetic foot ulcers, skin infections and burn wounds in clinic traits (Browne et al., 2020; Mahlapuu et al., 2020), and several researches have reviewed their topical application in wound healing (Dart et al., 2019; Nguyen et al., 2020; Thapa et al., 2020). Herein, we first reviewed the topical application of AMPs for bone repair (Figure 1). In this review, diverse structures of AMPs are initially summarized and additional focuses are laid on those in clinic traits. Then potential effects of AMPs for bone tissue engineering are presented, which include bactericidal activity, immunomodulation and regenerative effects. And we also summarize topical strategies to immobilize or deliver AMPs for bone repair. The motivation of this review is to successfully promote the clinical translation of AMPs for bone tissue engineering.

## 2 Diverse antimicrobial peptides with distinctive structures

**FIGURE 2:**
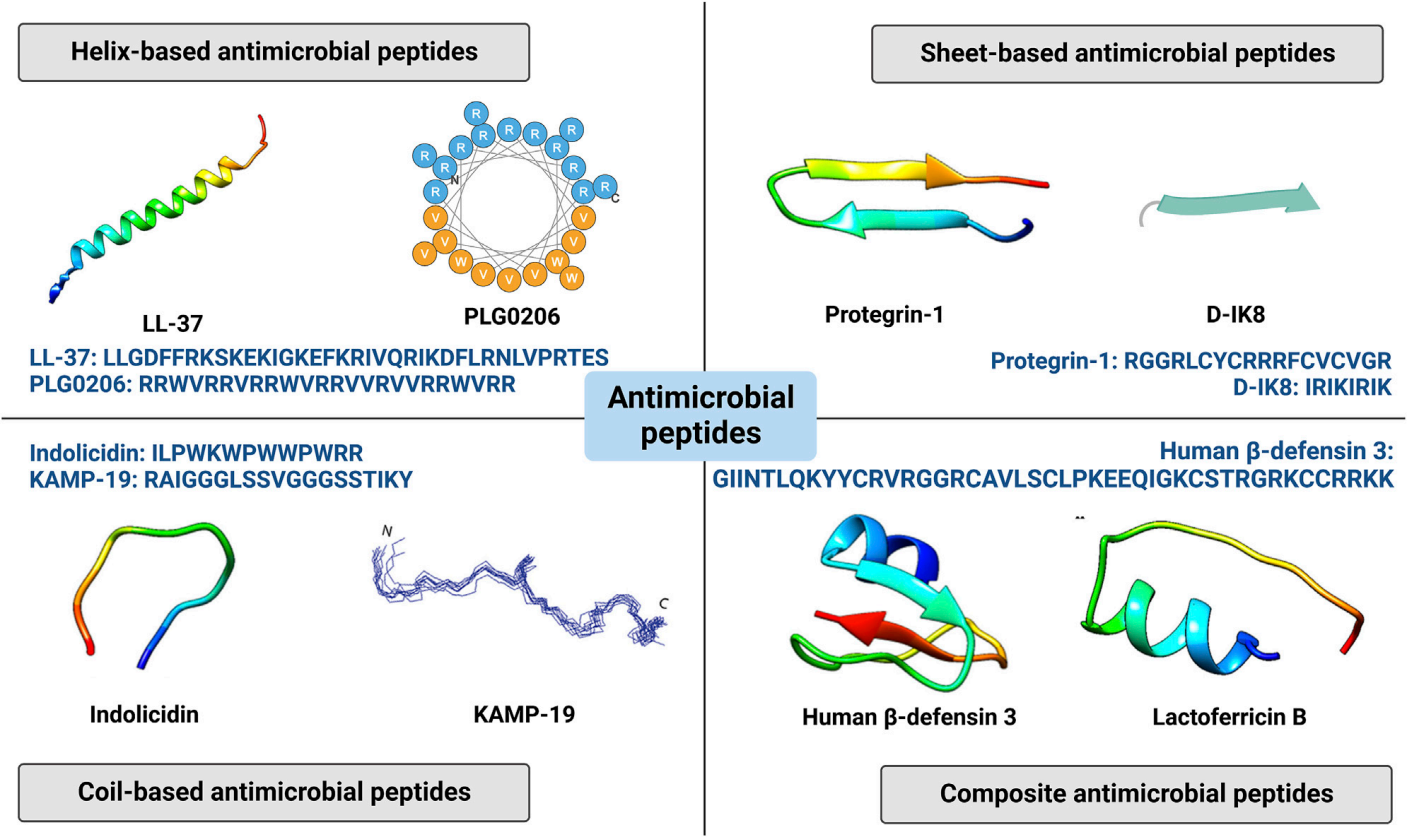
Four categories of antimicrobial peptides (helix based, sheet-based, coil-based and composite antimicrobial peptides) and related representative structures and sequences. Reprinted with permission from Di Somma et al. (2020). (Copyright 2020; Biomolecules), Huang et al. (2021). (Copyright 2021; Antibiotics), and Lee et al. (2016). (Copyright 2020; Frontiers in microbiology).

AMPs are a series of oligopeptides with 5–100 amino acids, charactered by a cationic character with net charge from +2 to +13, hydrophobicity with generally 50% hydrophobic resides, and amphipathicity which regulate the balance between hydrophilic residues and hydrophobic resides (Kumar et al., 2018). They can be obtained from virtually all organisms including bacteriophages, bacteria, fungus, plants and animals (Bin Hafeez et al., 2021). Depending on their major secondary structures, AMPs can be mainly divided to four groups: helical AMPs, sheet-based AMPs, extended AMPs, and composite AMPs (Figure 2). And some AMPs may further show cyclic structures in head-to-tail, side chain-to-tail, head-to-side chain or side chain-to-side chain models (Chow et al., 2019).

### 2.1 Helix-based antimicrobial peptides

Many kinds of helical structures exist in nature, such as α-helix, collagen triple helix and β-spinal helix. Most of helical AMPs are based on α-helix which generally has 3.6 amnio acids per turn. The α-helical structure is formed by hydrogen bonds, which are attributed to the interaction between the N-H group of the *n*th residues and the C=O group of (n-4)^th^ residues (Zou et al., 2020). And those redisues that rarely induce steric interference are typically used to construct α-helix, so α-helicalAMPs are rich in Leu, Ala, Gly, and Lys (Bin Hafeez et al., 2021). Common α-helical AMPs mainly include LL-37, magainin, melittin, cecropin, pleurocidin, moricin, aurein1-2, brevinin 1, maculatins, citropin, and buforin (Zou et al., 2020; Bin Hafeez et al., 2021). And some analogs derived from them have been tested in clinical trials.

LL-37 (LLGDFFRKSKEKIGKEFKRIVQRIKDFLRNLVPRTES) is an α-helical AMP with 37 amino acids obtained by proteolytic cleavage from the C-terminal of a human cathelicidin antimicrobial protein named hCAP18, it and its derivatives have been used in clinical trials. Showing +6 net charge under physiological pH, LL-37 has multiple functions for tissue repair including antibacterial and antibiofilm properties, immunomodulation, and angiogenesis. In a phase 2 clinical trial, LL-37 has been topically loaded to cream for the treatment of diabetic foot ulcer (NCT04098562). Another phase 2 clinical trial also explored its therapeutic effects in hard-to-heal venous leg ulcers (EudraCT 2018-000536-10). However, there are mainly three disadvantages corresponding to LL-37: high cost for synthesis because of long sequence, rapid protease degradation and existing cytotoxicity and hemolysis (Ridyard and Overhage, 2021). So dozens of LL-37 derivatives have been developed, among which OP-145 (IGKEFKRIVERIKRFLRELVRPLR) has been used to treat chronic suppurative otitis media in clinic trail (ISRCTN12149720).

Pexiganan (GIGKFLKKAKKFGKAFVKILKK) is a synthetic analog derived from magainin (GIGKFLHSAGKFGKAFVGEIMKS), an α-helical AMP with 23 residues in length which is identified from the African clawed frog (*Xenopus laevis*) (Goldstein et al., 2017). The AMP has been tested in several clinical trials. Lipsky et al. compared the treatment efficacy for infection between topical pexiganan cream and systemic oral ofloxacin in patients with mildly infected diabetic foot ulcer, and found that topical pexiganan showed equivalently effective results with oral ofloxacin of clinical improvement rates, microbiological eradication, and wound healing, but without apparent bacterial resistance (NCT00563394 and NCT00563433) (Lipsky et al., 2008). Besides, another group further compare the antimicrobial treatment for mild infections of diabetic foot ulcers between topical pexiganan cream and placebo cream in two phase 3 clinic trails (NCT01590758 and NCT01594762).

Showing an optimized amphiphilic αhelical structure, PLG0206 (RRWVRRVRRWVRRVVRVVRRWVRR) which was firstly named as WLBU2 is another synthetic AMP derived from lentivirus lytic peptide 1, which contains a hydrophilic surface composed of arginine residues and a hydrophobic surface containing valine and tryptophan residues (Deslouches et al., 2005). The AMP has been confirmed to highly target to bacteria with limited toxicity, to reduce biofilm burdens, and to show broad-spectrum antimicrobial activity (Huang et al., 2021). In a phase 1 clinical trial, it has been revealed that the AMP is safe and well tolerated by healthy research objects when it is intravenously infused with doses from 0.05 to 1 mg/kg (Huang et al., 2022). In another clinical trial, PLG0206 have been intravenously tested at 3 mg/kg for microbial infections. (ACTRN12618001920280).

In addition to α helix, other helical structures may also exist in AMPs. For example, PR-39 (RRRPRPPYLPRPRPPPFFPPRLPPRIPPGFPPRFPPRFR) is a proline rich AMP with proline residue accounting for almost half of the amino acid sequence. Although proline residue disturb the formation of α-helix, it was shown that the peptide contains collagen like type II poly-l-proline helix conformation (Holani et al., 2016).

### 2.2 Sheet-based antimicrobial peptides

Multiple sheet-based structures exist in nature, including parallel β-sheet, anti-parallel β-sheet, and β-hairpin. Most of sheet-based AMPs adopt a β-hairpin like conformation, which contain conserved cysteine residues to form disulfide bonds (Bin Hafeez et al., 2021). Common sheet-based AMPs include protegrins, bactenecin, defensins, tachyplesins, and polyphemusin, and some of their analogs have been used in clinical trials.

Most β-hairpin AMPs and derivatives are isolated or modified from natural AMPs. Protegrin-1 (RGGRLCYCRRRFCVCVGR) isolated form porcine leukocytes is one of β-hairpin like antimicrobial peptides with broad-spectrum antimicrobial activity (Steinberg et al., 1997). Derived from protegrin-1, iseganan and murepavadin have been used in clinal trials. Iseganan, also named as IB-367, has been topically used in oral solution to prevent oral infections after chemotherapy or radiotherapy (Giles et al., 2003; Trotti et al., 2004) Elad et al. (2012). Found that topical application of iseganan in patients suffered from chemotherapy could dramatically lower the loads of oral aerobic bacterial, streptococcal, and yeast. Murepavadin (POL7080) is another protegrin-1 analog, which shows potent antimicrobial activity to extensively drug resistant (XDR) *Pseudomonas aeruginosa* isolated from clinic patients (Sader et al., 2018). Intravenous murepavadin has been tested in clinic trials for the treatment of hospital-acquired bacterial pneumonia (HABP) and ventilator-associated bacterial pneumonia (VABP) caused by *Pseudomonas aeruginosa* (Martin-Loeches et al., 2018). And it has been shown that intravenous murepavadin was well tolerated in healthy subjects and subjects with renal function impairment (Dale et al., 2018; Wach et al., 2018). Bactenecin (RLCRIVVIRVCR) is also a sheet-based AMP showing β-hairpin conformation with a disulfide bond, which shows broad antimicrobial activities (Lee et al., 2008). Sun et al. used strategies of amino acid substitutions to improve net cationic charge and lower hydrophobicity, thus designing six derivatives of bactenecin: RRFRIVVIRWLR, RRWRIVVIRWRR, RKWRIVVIRVRR, RWRRIVVIRVRR, RWKRIVVIRKRR, and RKRRIVVIRRKR. And they found that these derivatives, especially RKWRIVVIRVRR and RWRRIVVIRVRR, showed increased antimicrobial activities and reduced cytotoxicity when compared with bactenecin.

Some β-hairpin like AMPs can be rationally designed depending on the design principles of β-hairpin supramolecular peptides, which could assemble to form nanofiber matrices and show antimicrobial activities. Based on the design principle of β-hairpin supramolecular peptides, Veiga et al. (2012). Designed an arginine-rich AMP named PEP8R (VRVRVRVRV^D^PPTRVRVRVRV), and they found that hydrogels assembled by PEP6R at 1.5% or higher concentrations show potent antibacterial activities towards *Staphylococcus aureus (S. aureus), Escherichia coli (E. coli)* but with great cytocompatibility. And the difference between β-hairpin like AMPs with both antimicrobial properties as well as assembling properties and those without assembling properties lies in the former follow the structure feature that hydrophobic residues and hydrophilic residues are regularly spaced except some residues to form β-turn structures.

In addition to those AMPs adopting β-hairpin conformation, there are also some iterative β-sheet AMPs. D-IK8 (IRIKIRIK) is an iterative ion-complementary β-sheet AMP composed of D-amino acids, which show enhanced protein stability, increased antimicrobial activities and unchanged hermetic properties when compared with its L-enantiomer (Ong et al., 2014). And it has been revealed that D-IK8 show strong activities against methicillin-resistant *Staphylococcus aureus* (MRSA) (Mohamed et al., 2016). Besides, Jiang et al. (2015). Designed a series of multidomain peptides assembling in β-sheet conformations: K_2_W(QL)_6_K_2_, WK_2_(QL)_6_K_2_, and K_3_W(QL)_6_K_2_, and found that the solution of K_3_W(QL)_6_K_2_ showed the best antimicrobial activities with MIC of 20 μM. But when these peptides assembled into nanofiber hydrogels at 2 wt% concentration, K_3_W(QL)_6_K_2_ hydrogels with worst mechanical properties demonstrated the lowest antimicrobial properties while K_2_W(QL)_6_K_2_ with highest storage modules showed the best bactericidal effect. These results revealed that the antimicrobial effects of hydrogels formed by sheet-based AMPs are determined by surface chemistry and mechanical properties of nanofiber hydrogels.

### 2.3 Coil-based antimicrobial peptides

Coil-based AMPs present unique structures devoid of both α-helical and β-sheet structures. And these peptides are generally rich in tryptophan, proline, and glycine (Bin Hafeez et al., 2021). Indolicidin (ILPWKWPWWPWRR), which belongs to the cathelicidin family, is one coil-based AMP rich in tryptophan and proline, and the AMP shows high potency against various microorganisms including bacteria, fungi and viruses (Batista Araujo et al., 2022). To improve bioactivity and reduce cytotoxicity, multiple derivatives have been developed, among which omiganan (ILRWPWWPWRRK) has been topically tested in clinic studies. In a randomized controlled trial, topical omiganan gel has been verified to reduce papillomavirus load in patients with external anogenital warts (Rijsbergen et al., 2020). Two clinic research GL13K explored the effects of topical omiganan gel for atomic dermatitis patients, and the results revealed that the AMP could reduce *Staphylococcus* genus and improve microbial diversity (Niemeyer-van der Kolk et al., 2020; Niemeyer-van der Kolk et al., 2022). And omifanan was also tested in other clinic diseases such as atheter infections (NCT00231153) and acne vulgaris (NCT02571998) (Mahlapuu et al., 2020). Besides, KAMP-19 (RAIGGGLSSVGGGSSTIKY) derived from keratin is another coil-based AMP, which has been verified to adopt a random coil structure in the presence of membrane mimicking sodium dodecyl sulfate micelles or lipopolysaccharides/1,2-dielaidoyl-sn-glycero-3-phosphoethanolamine liposomes. And it was also shown that the peptide could exert bactericidal effects by inducing pore forming and destruct bacterial cell membrane (Lee et al., 2016).

### 2.4 Composite antimicrobial peptides

While abovementioned AMPs mainly show a single secondary structure, there are a plethora of AMPs presenting multiple structures, which can be assumed as composite AMPs. Some natural AMPs contains multiple structures. For example, human β-defensin3 (HBD-3), GIINTLQKYYCRVRGGRCAVLSCLPKEEQIGKCSTRGRKCCRRKK, is a canonic example which presents αβββ conformations (Dhople et al., 2006). Cystatin (SEDRSRLLGAPVPVDENDEGLQRALQFAMAEYNRASNDKYSSRVVRVISAKRQLVSGIKYILQVEIGRTTCPKSSGDLQXCEFHDEPEMAKYTTCTFVVYSIPWLNQIKLLESKCQ) is also a naturally existing AMP containing both α-helix and β-sheet structures. Moreover, multiple structures can be also endowed to AMPs by artificially splicing distinctive peptide pieces from different agents. Melimine (TLISWIKNKRKQRPRVSRRRRRRGGRRRR) is a synthetic AMP which combines functional regions of mellitin (an α-helical AMP with 26 residues obtained from the venom of European honey bee) and protamine, which shows broad spectrum antibacterial properties (Yasir et al., 2020). Dutta et al. covalently coated melamine to contact lenses which were used in a 1-day clinical trial, and found that the melamine-immobilized lenses shows high antimicrobial activity but without cytotoxicity. And it has been orally administrated for the treatment of keratitis in another clinical trial (ACTRN12613000369729). Furthermore, Mel4 (KNKRKRRRRRRGGRRRR) is a clipped AMP derived from melamine, which was also covalently loaded to contact lenses for extended wear in a clinical trial (Kalaiselvan et al., 2022). The results showed that Mel4 coated lenses failed to change the conjunctival microbiota compared with control lenses (Kalaiselvan et al., 2022). But it may retain antibacterial activity during the initial wearing since the long wearing time.

## 3 Potential effects of antimicrobial peptides for bone tissue engineering

**FIGURE 3:**
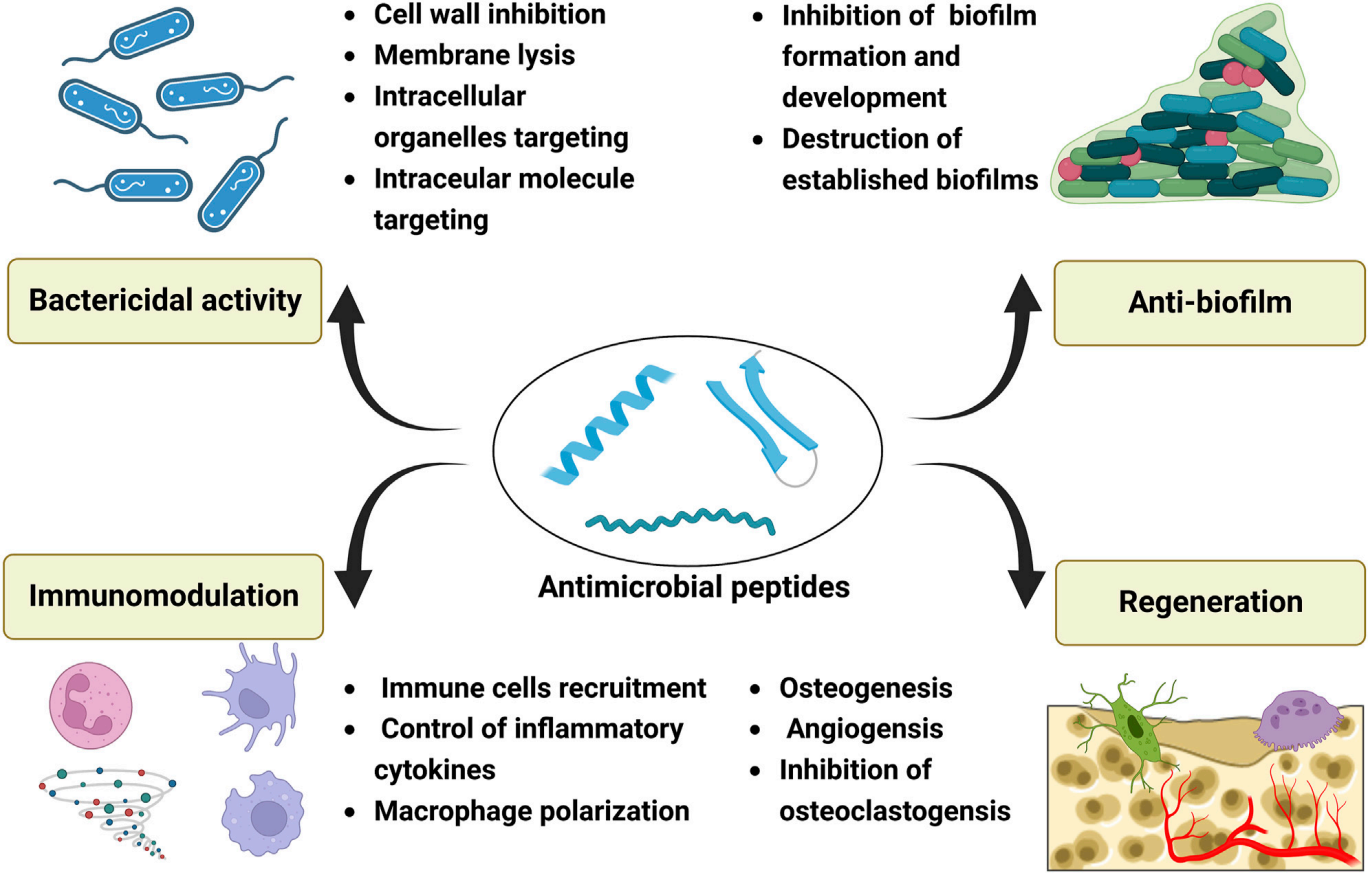
Potential effects of antimicrobial peptides for bone tissue engineering, and these effects can be mainly divided into four groups: bactericidal activity, anti-biofilm properties, immunomodulation and regenerative functions. Created with BioRender.com.

While most AMPs are used for soft tissue repair in clinic trials, they also show great promise for bone tissue engineering. They may exert their effects for bone repair by bactericidal activity, anti-biofilm formation, immunomodulation, and regenerative functions (Figure 3).

### 3.1 Bactericidal activity

One of critical effects of AMPs is bactericidal activity, and the antimicrobial mode of actions can be classified to two types: on the one hand, the integrity of bacterial structures such as cell membrane and cell wall may be directly disrupted by AMPs; on the other hand, AMPs could directly induce cell process inhibition by binding to specific molecules.

#### 3.1.1 Cell structure targeting

**FIGURE 4:**
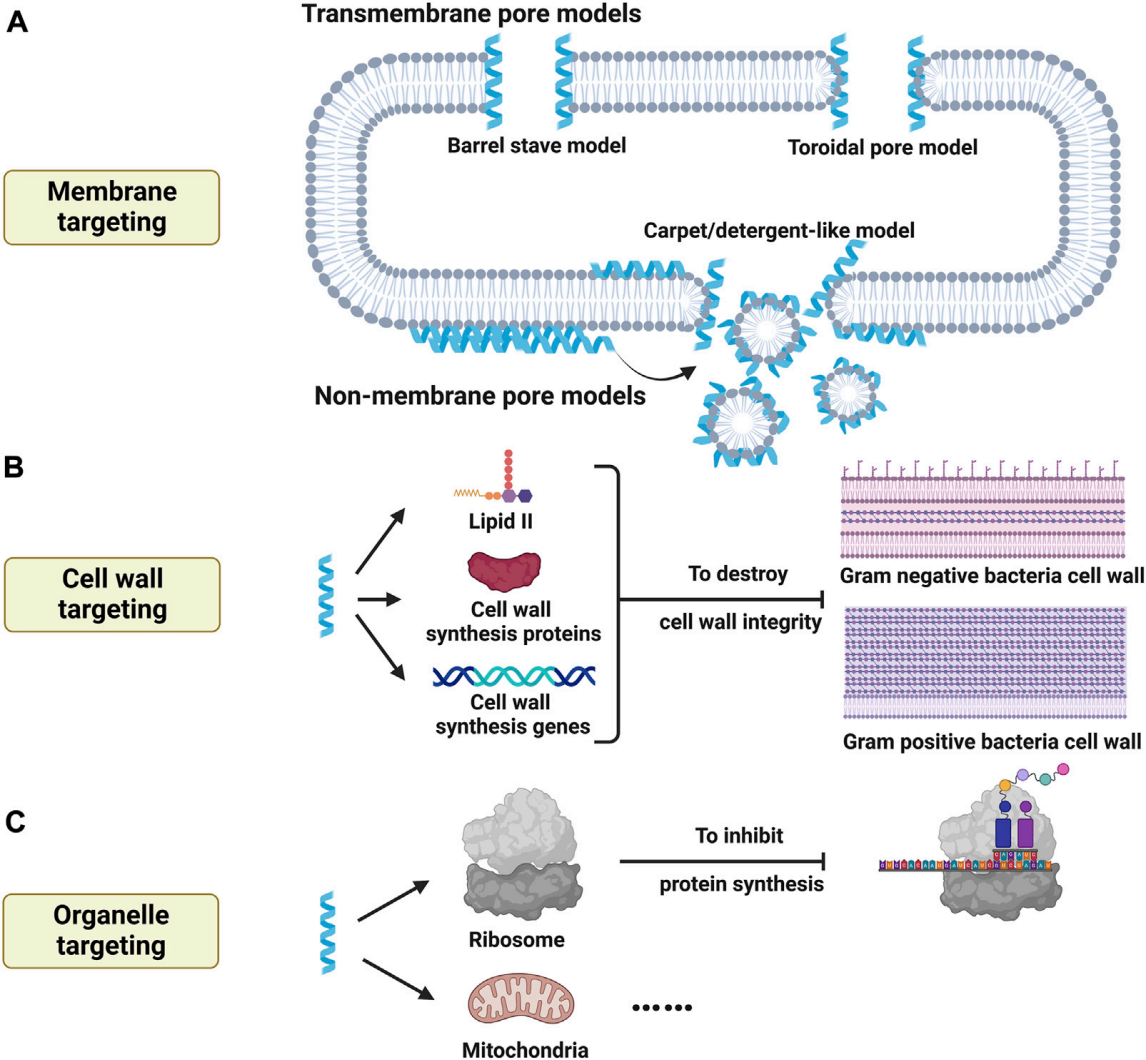
Bactericidal activity of antimicrobial peptides by targeting cell structures. **(A)** Antimicrobial peptides target to cell membrane to induce membrane lysis by transmembrane pore models (barrel stave model, toroidal pore model, *etc.*) and non-membrane pore models (Carpet/detergent-like model, *etc.*). **(B)** Antimicrobial peptides target cell wall synthesis and influence cell wall integrity by binding to lipid II, modulating cell wall synthesis related proteins and genes. **(C)** Antimicrobial peptides target organelles to influence physiological activities of microorganisms, thus exerting bactericidal effects. Created with BioRender.com.

The integrity of cell membrane is critical for the bacterial homeostasis, and most AMPs exert their antimicrobial effects by destabilizing bacterial membrane which is negatively charged because of anionic lipids such as lipopolysaccharides for Gram-negative bacteria or teichoic acids for Gram-positive bacteria. Animal cell membrane, in contrast, is composed of zwitterionic phospholipids such as phosphatidylcholine and sphingomyelin. So initial electrostatic interaction is generally initiated between anionic bacterial membrane and cationic AMPs. When AMPs aggregate on the surface of bacterial membrane to form stable conformation, two broad modes of action have been proposed to interpret antimicrobial mechanisms: transmembrane pore models and non-membrane pore models (Figure 4A) (Luo and Song, 2021).

Transmembrane pore models refer to that pores can be induced by AMPs within bacterial membrane. Barrel stave model is one of critical transmembrane pore models. When AMPs gather upon bacterial membrane above critical concentration, they cause membrane thinning and lipid shift after conformational changes, which then insert vertically to membrane bilayer with their hydrophobic regions towards hydrophobic regions of membrane liquids and their hydrophilic regions inside to form a channel lumen (Luo and Song, 2021). And the lateral interaction of barrel stave channels is enhanced by peptide-peptide interactions. Different form barrel stave model, toroidal pore model is another important transmembrane pore model. When AMPs aggregate at specific concentration, they mediate local curvature of membrane phospholipid, which cause the rearrangement of membrane bilayer. And AMPs interact with lipids to form toroidal pore channels but without lateral peptide-peptide interactions. Namely, the membrane layer is curved but not shifted which allows to form channels partly by AMPs and partly by phospholipid head group. Furthermore, there are also other transmembrane pore models such as disordered toroidal pore model and aggregate model (Nguyen et al., 2011).

Non-membrane pore models are termed that AMPs fail to induce pore formation but promote the formation of micelles. Carpet/detergent-like model is one of canonic non-membrane pore models. Initially, AMPs aggregate parallelly on the outer surface of bacterial membrane and interact with anionic phospholipids to form carpet structures. When the local concentration of AMPs reaches over a threshold concentration, bacterial membrane interacted with carpet can be further disintegrated to form micelles, which is known as a detergent-like effect (Nguyen et al., 2011). Agglutination model, besides, is also anther non-membrane pore model, and micelles are induced by the interaction between AMPs and cell wall components on the outer surface of bacterial membrane (Sinha et al., 2017). Moreover, other non-membrane pore models include but not limit to membrane thinning/thickening model and non-bilayer intermediate model.

While most AMPs show their antimicrobial activity by membrane lysis, some AMPs exert their antibacterial activity by influencing cell wall synthesis to destroy cell wall integrity (Figure 4B). Lipid II is an important precursor for cell wall synthesis, and some AMPs such as α-defensin Human Neutrophil Peptide 1 (HNP-1) show high affinity to this molecule and affect cell wall synthesis, which then undermines cell wall integrity (de Leeuw et al., 2010). Besides, Wenzel et al. (2014). Reported that a short AMP, RWRWRW-NH2, could insert into Gram-positive membrane and delocalize peripheral membrane proteins which are critical for cell wall synthesis, which then disturb cell wall integrity. Furthermore, cell wall synthesis can be also affected by the genetic modulating of AMPs. For example, it has been found that LL-37 could regulate *Sfp1* gene to affect cell wall synthesis, thus destabilizing cell wall intensity (Hsu et al., 2021). Another group also reported that cell wall intensity was damaged by a AMP, combined with upregulated cell wall synthesis-related gene and downregulated cell membrane ergosterol synthesis-related genes (Ma et al., 2020). Similar results were observed by a novel AMP named sparamosin26−54 (Chen Z et al., 2021). Therefore, cell wall integrity can be disturbed by influencing cell wall synthesis, and related mode of actions can be grouped to binding to cell wall precursor, modulating cell wall s1ynthesis proteins, and regulating cell wall synthesis related genes. And detailed modes of action are highly needed to interpret.

In addition to targeting membrane and cell wall, some AMPs may also target intracellular organelles (Figure 4C) Graf and Wilson (2019). Showed that proline-rich AMPs target ribosome and then inhibit protein synthesis to induce bacterial death. Besides, it has been also reported that an AMP named myristoyl-CM4 could target mitochondria of cancer cells and cause mitochondrial dysfunction, which then induce cancer cell death (Li C et al., 2018). But whether AMPs could target microbial mitochondria remains unknown, which need further researches.

#### 3.1.2 Intracellular molecule targeting

While most AMPs show their antimicrobial activity by destructing cell structures, especially membrane lysis, some AMPs also target intracellular molecules to kill microbes after translocation. Nucleic acid is one of common molecular targets for AMPs. Buforin II is one AMP showing broad spectrum antimicrobial properties, which mainly binds to DNA and RNA to disturb cell functions but without membrane lysis (Park et al., 1998). In addition to direct binding, an AMP named TO17 have been reported to induce the degradation of genomic DNA and total RNA (He et al., 2018a). Proteins, besides, are also important intracellular targets for AMPs. As an example, Chung et al. revealed that LL-37 could translocate bacterial membrane and bind to *Francisella* cytoplasmic acyl carrier protein (AcpP), which then changed the profiles of fatty acid (Chung et al., 2015). Moreover, AMPs could also target intracellular enzyme to exert anti-microbial functions. One study from Braffmana’s group revealed that AMPs microcin J25 (MccJ25) and capistruin (Cap) could bind to the second channel of RNA polymerase and inhibit its bioactivity (Braffman et al., 2019).

### 3.2 Anti-biofilm properties

**FIGURE 5:**
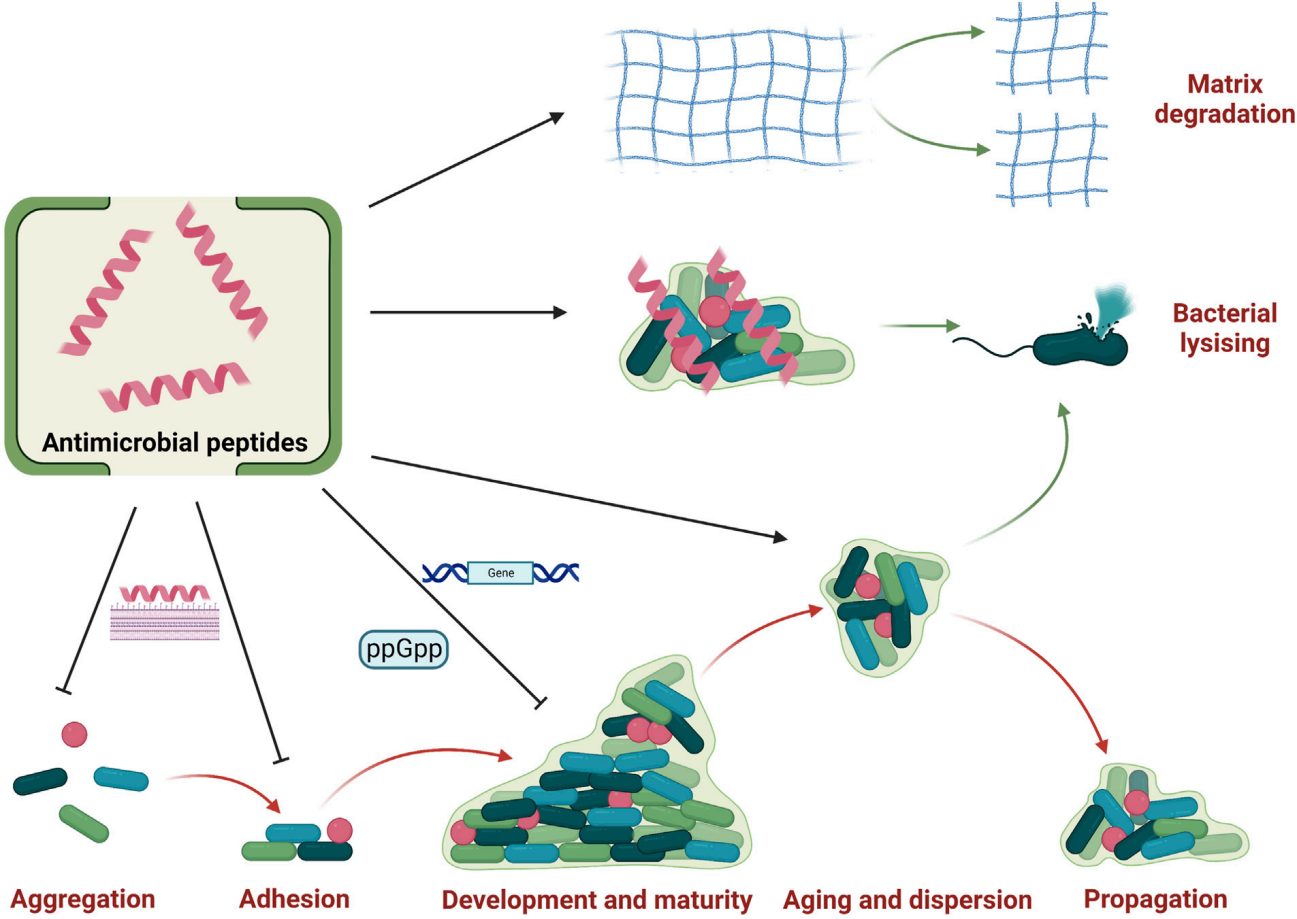
Anti-biofilm properties of antimicrobial peptides. Antimicrobial peptides could influence the biofilm cycle by inhibiting aggregation and adhesion as well as restraining the development and maturity. They also promote biofilm aging and dispersion, thus exposing bacteria to antimicrobial peptides to induce bacterial lysis. Besides, some antimicrobial peptides could also promote the matrix degradation of established biofilm. And there are also some antimicrobial peptides could penetrated established biofilm and interact with bacteria to exert bactericidal activity. Created with BioRender.com.

Biofilms are sessile microbial communities which are attached to a biotic/abiotic surface or embedded in a matrix as aggregates (Roy et al., 2018). The surface of biomaterials is vulnerable to support the formation of biofilm when bone repair materials are implanted to bone defects. And biofilms are generally correlated to chronic bone infection, which are difficult to treat because of bacterial resistance (Vallet-Regí et al., 2020). So anti-film properties are highly needed for bone tissue engineering. And recent studies showed that some AMPs show potent anti-film properties (Figure 5).

Some AMPs could inhibit biofilm formation and development. Four stages are generally needed for biofilm formation and development: microorganism aggregation or attachment, microbial adhesion, biofilm development and maturity, and biofilm aging (Di Somma et al., 2020; Luo and Song, 2021). Inhibiting initial attachment is one of important mode of action for AMPs to obstacle biofilm formation. For example, LL-37 have been reported to bind to the main cell wall component (mannan) of *Candida albicans* to inhibit initial adhesion, which may help to inhibit biofilm formation (Tsai et al., 2011). Another research also showed that a short AMP 1037 with nine residues could obstacle initial attachment by suppressing swimming and swarming motilities (de la Fuente-Núñez et al., 2012). Besides, AMPs could exert anti-biofilm functions by modulating guanosine 5′-diphosphate 3′-diphosphate (ppGpp) and guanosine 5′-triphosphate 3′-diphosphate (pppGpp) which are two critical molecules for biofilm formation and development in alarm system to encounter stress environments. As an example, an AMP 1018 has been reported to target enzymes RelA and SpoT to block the synthesis of ppGpp and pppGpp, thus showing potent anti-biofilm properties (de la Fuente-Núñez et al., 2014). Furthermore, AMPs also induce genic regulation to inhibit biofilm formation and development. The AMP 1037 has been reported to downregulate or upregulate some gene expressions to inhibit biofilm formation and development (de la Fuente-Núñez et al., 2012). Besides, it was also reported that AMPs could promote biofilm aging and dispersion, which promote AMPs to kill bacteria (Hancock et al., 2021).

Some AMPs could destruct established biofilms, and two main modes of actions have been proposed. On the one hand, some AMPs could directly induce the degradation of biofilm matrix. Libardo et al. reported that an AMP piscidin three from fish could interact with Cu^2+^ to activate nuclease activity to clear extracellular DNA, thus damaging pre-formed biofilms (Libardo et al., 2017). On the other hand, some AMPs could infiltrate biofilms and destroy bacterial membrane in biofilms. One study showed that an AMP sculentin (1–21) could disturb membrane of *pseudomonas aeruginosa* in biofilms, thus induce biofilm disruption (Luca et al., 2013).

### 3.3 Immunomodulation

In addition to direct anti-microbial and anti-biofilm properties, AMPs could modulate immune system to enhance microbe clearing and control diverse inflammation. Both innate and adaptive immune system can be influenced by AMPs, the actions of which mainly include recruitment of multiple immune cells (such as macrophages, neutrophils, mast cells, dendritic cells, and lymphocytes), modulation of neutrophil functions, initiation of antigen-presenting cells, and activation of T cell/B cell responses (Mookherjee et al., 2020). And AMP-related immunomodulation mechanisms can be attributed to their interaction with membrane receptors (such as CXCR2 and FPR2) and intracellular receptors (such as SQSTM1 and GAPDH) of immune cells (Hilchie et al., 2013). Besides, AMPs could also control diverse inflammation by inhibiting pro-inflammatory cytokines and promoting anti-inflammatory cytokines, and related mechanisms mainly include intercepting inflammatory inducers to target to related sensors and suppressing inflammation-related signaling pathways and transcription factor expression (van der Does et al., 2019).

AMPs may promote bone tissue regeneration by immunomodulation, which mainly depends on macrophage polarization. Moderate macrophage polarization from M1 phenotype to M2 phenotype helps to bone tissue regeneration (Hao et al., 2022). And some AMPs have been verified to promote macrophage polarization. For example, Chen et al. (2018). Reported that an AMP cathelicidin-WA could effective promote M1 macrophages induced by *E. coli* K88 to switch to M2 macrophages. Another group also showed that an AMP, innate defense regulator-1018, could effectively transfer M1 state to M2-M1 state, which shows both anti-inflammatory and pro-inflammatory properties (Pena et al., 2013).

### 3.4 Regenerative functions

In recent years, AMPs have been verified to exert regenerative functions for bone tissue engineering by promoting angiogenesis and osteogenesis as well as inhibiting osteoclastogenesis. Angiogenesis is an important process during bone regeneration, which support the delivery of nutrients and oxygen. Some AMPs could induce angiogenesis. Histatin-1 is an AMP with 38 amino which is rich in human saliva, and the peptide have been shown to enhance angiogenesis, as confirmed by tube formation assay and the chick chorioallantoic membrane model (Torres et al., 2017). Besides, some AMPs could promote osteogenesis by modulate stem cell behaviors. LL-37 is a promising AMP to promote the proliferation, migration, and osteogenic differentiation of MSCs (Liu H et al., 2018). And human β defensin 3 has also been reported to promote MSC osteogenesis (Wang B et al., 2022). Furthermore, some AMPs could also inhibit osteoclastogenesis. Zhou et al. immobilized a AMP named GL13K (GKIIKLKASLKLL) to titanium surface, which was then used to culture RAW 264.7 cells, and they found that GL13K modified implant induced less osteoclast number than pristine implant (Zhou et al., 2021). Liu et al. also reported that LL-37 could reduce LPS-induce osteoclastogenesis (Liu Z et al., 2018).

## 4 Topical applications of antimicrobial peptides for bone tissue engineering

**FIGURE 6:**
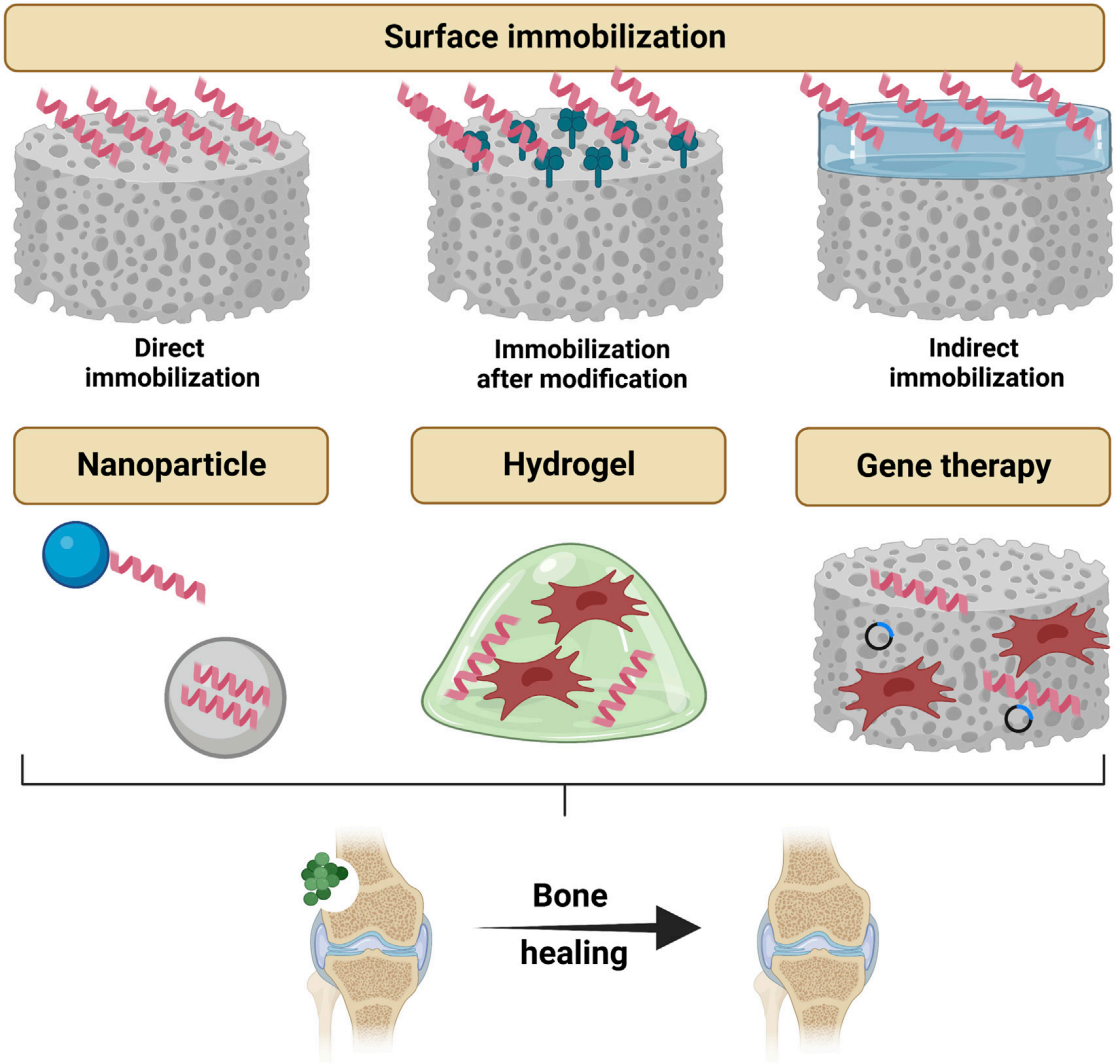
Topical strategies to administrate antimicrobial peptides for bone tissue engineering, which include delivery by surface immobilization (direct immobilization, immobilization after modification and indirect immobilization), delivery by nanoparticles, delivery by hydrogels and gene therapies. Created with BioRender.com.

Severe bone defects caused by trauma, infection and tumor formation often require surgical reconstruction using artificial implants (Panayotov et al., 2016). After the implantation procedure, pathogens tend to accumulate on the surface of the implant material, forming a biofilm that protects them from the host’s defenses (Ghigo 2001). Conventional implant materials are prone to induce biomaterial-associated infections (BAI) because they lack antimicrobial properties (Campoccia et al., 2013a). Once BAI occurs, it can lead to serious consequences, including surgical failure, sepsis, disability and even death (Campoccia et al., 2013b). In addition to ensuring as much asepsis as possible during the procedure, the prevailing clinical practice is the postoperative systemic prophylactic application of antibiotics for this nasty situation. However, systemic administration has problems such as low efficiency, local tissue toxicity, and the development of drug resistance (Dik et al., 2018). Therefore local application of antimicrobial agents is considered to be a more effective strategy for the prevention of BAI. AMPs have a unique bactericidal mechanism that significantly reduces the possibility of bacterial resistance (Wang et al., 2019). Thus AMPs are gradually replacing antibiotics as the active factor in such antimicrobial strategies. In this part, we summarize AMPs delivery strategies that may be applied to bone tissue engineering for subsequent investigators to develop more effective bone tissue engineering scaffolds (Table 1), and we also focus on potential value of AMPs to be used in gene therapy (Figure 6).

**TABLE 1:** Comparison of antimicrobial peptide properties and related applications.

AMPs	Structures	Delivery system	Antibacterial properties	Other properties	Applications	Ref
LL37	Helix-based	Surface immobilization, nanoparticles	*Staphylococcus aureus, methicillin-resistant Staphylococcus aureus (MRSA)*	Osteogenesis, Osteoclastogenesis inhibition, immunomodulation	Rat femoral defect model, rat infected bone defect model	He S et al. (2018)
						He et al. (2019)
						Shen et al. (2019)
LF1-11	Helix-based	Surface immobilization	*Streptococcus sanguinis, Staphylococcus aureus*	-	Antimicrobial and osteogenic assays *In vitro*	Hoyos-Nogués et al. (2017)
KR-12	Helix-based	Surface immobilization	*Pseudomonas aeruginosa, Staphylococcus aureus, Escherichia coli*	Osteogenesis	Rat femoral defect model	Meng et al. (2020)
						Trzcińska et al. (2020)
GL13K	Sheet-based	Surface immobilization	*Pseudomonas aeruginosa, Escherichia coli, Fusobacterium nucleatum, Porphyromonas gingivalis*	Osteoclastogenesis inhibition, immunomodulation	Rabbit femoral defect model	Chen J et al. (2020)
						Cheng et al. (2017)
						Fischer et al. (2021)
						Li et al. (2017)
HHC-36	Sheet-based	Surface immobilization, hydrogels	*Staphylococcus aureus, Staphylococcus epidermidis, Escherichia coli, Pseudomonas aeruginosa、MASA*	Osteogenesis	Rabbit tibial osteomyelitis model	Chen X et al. (2020)
						Cheng et al. (2017)
						Wang L et al. (2022)
Tet213	Coil-based	Surface immobilization, hydrogels	*Staphylococcus aureus, Pseudomonas aeruginosa*	-	Rabbit tibial osteomyelitis model	Kazemzadeh-Narbat et al. (2010)
						Yang et al. (2018)
Pac-525	Coil-based	nanoparticles	*Staphylococcus aureus, Escherichia coli, Porphyromonas gingivalis, Fusobacterium nucleatum, Streptococcus sanguis*	-	Antimicrobial and osteogenic assays *In vitro*	He et al. (2018a)
						He et al. (2020)
PSI 10	Coil-based	Surface immobilization, nanoparticles	*Escherichia coli, Staphylococcus aureus, MRSA*	-	Rat femoral defect model	Tian et al. (2015)
						Zhongxing et al. (2021)
HBD-3	Composite	Surface immobilization	*Staphylococcus aureus, Escherichia coli*	Osteogenesis	Antimicrobial and osteogenic assays *In vitro*	Liu H et al. (2018)
						Wang B et al. (2022)
MBD-14	Composite	Surface immobilization	*Staphylococcus aureus, Escherichia coli*	Osteogenesis	Rat femoral osteomyelitis model	Yuan et al. (2019)
Mel4	Composite	Surface immobilization	*Staphylococcus aureus, Pseudomonas aeruginosa*	-	Rabbit infected bone defect model	Zhang Q et al. (2021)

### 4.1 Delivery system for antimicrobial peptides

#### 4.1.1 Surface immobilization

Implant surface immobilization is an essential strategy in the field of antimicrobial delivery system, whereby coatings containing AMPs are loaded on the surface of the implant material to prevent biofilm formation at the tissue-material interface. Previous studies have developed a wide variety of AMP coatings. However, there are two main challenges in developing ideal AMP coatings: first, to immobilize them on the surface while ensuring their biological activity; and second, to load them at the right dose and release them at the right time. According to the position or order of the AMPs in the loading process, we classify the surface immobilization strategies of AMPs into three major categories: direct immobilization, immobilization after modification and indirect immobilization.

Direct immobilization means that the coating process does not involve any special treatment of the implant surface or the AMPs, and the peptide sequence is directly coated and immobilized on the surface of the implant material using a primary coating method. This is one of the simplest AMPs immobilization strategies. It directly co-incubates the AMPs solution with the implanted material, allowing the AMPs to physically adhere to the material surface. For example, Su et al. applied Mel4 solution directly to titanium scaffolds for investigating the effectiveness of AMPs in preventing infection after internal fixation of open fractures (Zhang S et al., 2021). They inoculated rabbits with *Staphylococcus aureus* and *Pseudomonas aeruginosa*, the common causative agents of open fractures, and implanted a titanium scaffold loaded with Mel4 into the rabbit femur to observe its therapeutic effect on postoperative infection. This simple titanium surface coating demonstrated significant antimicrobial efficacy, which provides a promising strategy for controlling infections after open fracture internal fixation. In addition, Li et al. immobilized the bifunctional chimeric peptide P15-CSP on the surface of tissue culture (TC) plastic by dry-coating, designing a bifunctional surface that is both antibacterial and osteogenic (Li et al., 2015). This chimeric peptide is a combination of the antimicrobial peptide P15 and the capacity-stimulating peptide (CSP) *via* the linker peptide A (EAAAK)nA, and both peptides are cationic peptides. In contrast, TC plastics have a special anionic surface, so they immobilize P15-CSP on the material surface by inter-ionic forces. Despite the initial success of these direct immobilization strategies for AMPs loading, two problems may be faced by simple physical adsorption alone: non-uniform loading of AMPs and poor immobilization to achieve long-term antimicrobial effects.

Immobilization after modification refers to the treatment of the implant surface or processing of the AMPs prior to immobilization. Common implant surface treatment measures include sulfonation, silylation, surface modification with polydopamine, and electrolytic deposition techniques. In addition, the pre-processing of AMPs mainly consists of linking metal-binding sequences or specific anchoring sequences. Compared to direct immobilization, modification followed by immobilization provides a uniform and stable fixation of the AMPs on the surface of the implant material. For example, Yuan et al. immobilized mouse beta-defensin-14 (MBD-14) on the surface of a polyetheretherketone (PEEK) bone scaffold to improve the antimicrobial activity and osseointegration of the scaffold (Yuan et al., 2019). Long-term drug delivery of MBD-14 is difficult to achieve by direct coating immobilization alone. Previous studies have shown that porous surfaces facilitate drug loading and delivery (Andrew et al., 2010; Lee et al., 2019). To increase the AMPs loading, they treated the PEEK surface by a sulfonation technique before immobilizing the AMPs. The PEEK surface was etched with concentrated sulfuric acid to create a three-dimensional porous structure, after which the AMPs was freeze-dried and immobilized on its surface. This MBD-14-loaded PEEK scaffold inhibited *Staphylococcus aureus* and *Pseudomonas aeruginosa* for up to 28 days in an *in vitro* antimicrobial assay. This excellent slow release effect resulted from the high AMPs loading due to the porous structure and the covalent binding of MBD-14 to the residual sulfonic acid groups on the surface of the sulfonated material. In addition, Kazemzadeh-Narbat et al. treated titanium plates by using electrolytic deposition technique to form microporous calcium phosphate coatings on their surfaces (Kazemzadeh-Narbat et al., 2010). It creates a larger surface area for the peptide to interact with the material surface. And the cationic antimicrobial peptides can be electrostatically attracted to the negatively charged phosphate groups in the calcium phosphate coating, enhancing the retentive effect while increasing the loading. In addition, the calcium phosphate coating has been shown to have osteoconductive properties that promote bone formation around the implant (Kazemzadeh-Narbat et al., 2010). This multi-effect scaffold is a desired characteristic for the ideal orthopedic implant.

Another popular material surface treatment strategy is silanization. Silanization is the process of surface treatment of metallic or non-metallic materials with an aqueous solution of organosilane to functionalize the surface. This method is commonly used to modify hydroxyl-rich materials, such as titanium or other metal oxides. Silylation enables the introduction of reactive groups, such as amino or carboxyl groups, making it possible to chemically graft AMPs on the surface of biologically inert materials. Due to the effectiveness and low cost of silylation technology, a large number of studies in the field of AMPs delivery have converged (Hoyos-Nogués et al., 2017; Chen J et al., 2020; Fischer et al., 2021). For example, Chen et al. immobilized the antimicrobial peptide GL13K on a titanium implant scaffold by silanizing the titanium surface with a CPTES solution (Chen X et al., 2020). Specifically, the free amine (nucleophile) of the peptide underwent a direct nucleophilic substitution reaction with the chlorine atom (leaving group) of the organic functional group of CPTES, resulting in the immobilization of AMPs by forming a covalent bond with the silanized surface (Fischer et al., 2021). The lysine terminus of GL13K is a potential anchor site, and previous studies have demonstrated that this anchor site does not affect its antimicrobial activity. Based on this, they safely and effectively loaded GL13K onto titanium-based scaffolds and demonstrated that the modified titanium implants inhibited peri-implantitis through immunomodulatory effects. Our previous literature has shown the important role of immune regulation in osteogenesis (Chen et al., 2022). Therefore, surface-functionalized scaffold materials with immunomodulatory properties are highly promising strategies in orthopedic repair. In addition, AMPs immobilized on the surface of the implant material inhibit the adhesion of bacteria on the surface of the material along with the adhesion of osteoblasts. This is certainly unfriendly to peri-implant osseointegration. For this reason, Hoyos-Nogués et al. coupled the antimicrobial peptide LF1-11 to the RGD peptide, a cell adhesion sequence, *via* a solid-phase synthesis platform and covalently grafted it onto the surface of titanium implants with the help of silylation techniques (Hoyos-Nogués et al., 2017). The equimolar coupling of two peptide sequences with different activities ensured uniform cell adhesion and antimicrobial potential. Antibacterial tests and osteogenic series showed that this modified titanium plate demonstrated good antibacterial properties along with improved osteoblast adhesion, proliferation and mineralization. This is an advantage over classical monofunctional bone scaffolds. In additional, some optimization strategies for oral implants using silanization technology to load AMPs are very novel. Since the challenges faced by oral implants and orthopedic implants are similar: antimicrobial and osseointegration, we believe that some loading strategies in the oral field can provide a reference for orthopedic implants. For example, Fischer’s team has produced a titanium surface coating with dual bone-enabling and antibacterial functions through silanization and peptide co-fixation. The coating is characterized by its bioreactivity. The scaffold is loaded with GL13K, which provides antimicrobial activity, and matrix metalloprotease 9 (MMP-9) cleavable peptide (MMP9-CP), which provides bone-enabling effects. Among them, MMP9-CP is an enzyme-mediated cleavable peptide that promotes the release of MMP9 in the presence of osteoclast-released MMP9. MMPs are known to play an important role in bone repair and bone remodeling (Sternlicht and Werb, 2001). Therefore, future bone tissue engineering scaffolds can learn from this multifunctional coating strategy with bioresponsiveness to play a precise role in the corresponding bone repair process. Although the chemical immobilization of AMPs on the surface by silane coupling agents has been very effective, the complex and time-consuming pre-treatment of the material and the potential cytotoxicity of the complex chemical process are issues that need to be considered when utilizing this immobilization strategy in the future (He et al., 2018b).

In addition, over the past decade or so, scientists have benefited from the revelation of mussel foot adhesion proteins to develop polydopamine-based coating strategies (Ryu et al., 2018). Specifically, because the quinine and catechol groups of polydopamine can create a chelated structure with the material through a series of chemical reactions, it has broad adhesion properties and can adhere to almost any surface. The mechanism by which polydopamine coatings can be used to load bioactive factors such as AMPs lies in the fact that such amine-containing nucleophilic reagents can be coupled to pDAs *via* Schiff base reactions or Michael addition reactions of benzoquinone groups under weak alkaline conditions, leading to secondary modification (Trzcińska et al., 2020). The advantages of the polydopamine coating strategy are low cost, ease of handling, wide application and optimized biocompatibility. So this strategy is also widely used in AMPs delivery system (He S et al., 2018; Meng et al., 2020; Trzcińska et al., 2020). For example, Trzcińska et al. (2020). Introduced an autonomously synthesized KR-12 analogue on the surface of titanium implants *via* a polydopamine coating. In detail, the treated titanium plates were first co-incubated with dopamine in alkaline Tris buffer to form a polydopamine coating on the titanium surface, providing an active platform for subsequent secondary modification of AMPs. AMPs solutions were then prepared at the MIC value of this peptide and co-incubated with pDA-coated titanium plates to obtain AMPs-loaded titanium implants (Trzcińska et al., 2020).5 (6)-FAM fluorescent labeling showed successful loading of AMP on the coating surface. In addition to the initial 6 h burst release, 50%–60% of the peptide remained unreleased after 30 days, indicating a very stable and reliable attachment of the lysine residue of the peptide to the dopamine surface and a reliable way to load AMPs on future orthopedic implant materials with slow local release. In addition, Meng et al. introduced KR-12 on the surface of PEEK bone implants using pDA as a linking layer and applied it to the repair of femoral defects in rats (Meng et al., 2020). The secondary modified PEEK-pDA-KR-12 scaffolds showed significant improvement in physical properties such as hydrophilicity as well as surface roughness. Furthermore, they immersed the prepared active scaffolds in *S. aureus* solution and implanted them into rat femurs after 24 h. The experiments showed that the scaffolds loaded with KR-12 had remarkable antibacterial activity (Meng et al., 2020). In addition, *in vivo* studies in mice demonstrated that the scaffold significantly promoted osteogenic differentiation of MSCs and peri-implant bone formation, which is consistent with previous reports. Previous reports have demonstrated that KR-12 promotes osteogenic differentiation of MSCs through the bone morphogenetic protein (BMP)/Smad signaling pathway without cytotoxicity (Li H et al., 2018). In conclusion, the loading of KR-12 through the PDA junction layer can improve the defect of low osseointegration ability of PEEK scaffolds and confer good antimicrobial activity. This approach can be applied to many other types of bone regeneration materials in the future.

In addition to treating the surface of the material, treating the AMPs themselves is a popular and effective loading strategy. Binding sequences are peptide sequences that are able to bind to specific surfaces or functional groups. Modifying AMPs sequences with specific binding sequences to give them a stronger binding ability to the surface is also a mainstream approach to immobilize AMPs on the surface of implant materials (Zhang et al., 2018; Wisdom et al., 2020; Zhongxing et al., 2021). The advantage of this loading strategy is that it does not change the surface of the material, thus avoiding the destruction of its original properties. Yazici et al. designed bifunctional peptides with titanium surface affinity and antimicrobial activity, TiBP1-GGG-AMP and TiBP2-GGG-AMP, based on titanium-binding sequences (Haynie et al., 1995; Yazici et al., 2016). This type of peptide is composed of a solid-binding peptide and an AMP motif (E14LKK) by standard solid-phase synthesis technique to chimerize. They use a triple glycine (GGG) flexible linker to couple the two peptides to ensure that the activity of both parts is not affected. It was shown that the two designed peptides have stronger affinity than simply direct immobilization. And *in vitro* experiments, the titanium plates coated with the chimeric peptides exhibited significant anti-biofilm properties against both Gram-positive and Gram-negative bacteria (Yazici et al., 2016). Wisdom et al. also used the peptide binding sequence to modify AMP and highlighted that the chimeric peptide could achieve 100% coverage of the titanium sample surface within minutes (Wisdom et al., 2020). This result further supports the advantages of solid binding peptides in the field of AMPs immobilization. Liu et al. used hydroxyapatite binding domain (HABD) coupled to bone morphogenetic protein two mimetic peptide (BMP2-MP) and antimicrobial peptide PSI10 (RRWPWWPWRR), respectively, to enhance their binding to HA bone scaffolds (Zhongxing et al., 2021). They fabricated HA bone repair scaffolds using 3D printing technology and compared the binding ability of chimeric peptides and common peptides to the surface of the material. The results showed that the chimeric peptide containing HABD adsorbed 9–11 times more than the unmodified peptide on the surface of the HA scaffold. So, HABD is a “bridge” between AMPs and the material surface, which can increase the drug loading and achieve the slow release of AMPs at the same time (Bansal et al., 2019). The antimicrobial assay suggested that HABD labeling did not affect the activity of the peptide. Although Liu did not integrate the osteogenic and antimicrobial active units into the same bone scaffold, this loading strategy provides clarity for the subsequent fabrication of osteogenic scaffolds. In addition to modification using the specific binding sequences mentioned above, non-specific binding sequences, such as DOPA sequences, have also been applied for peptide pretreatment. Li et al. chimerized DOPA sequences with AMP so that AMP could be anchored to the PEEK bone scaffold (Li et al., 2021). They constructed a rabbit infectious osteomyelitis model and implanted AMP-loaded PEEK scaffolds into rabbit femurs. The results showed that the scaffold had a long-term stable antimicrobial effect as well as optimized osseointegration ability. In conclusion, the key to AMPs by combining sequence modifications lies in how to ensure the function of each functional domain to the maximum extent possible. To cope with this problem, researchers usually utilize a variety of spacer units (rigid linkers, flexible linkers, *etc.*) as bridges for coupling (Yazici et al., 2016; Wisdom et al., 2020). As to which protection measures are the most effective remains to be further explored.

Indirect immobilization refers to the physical separation of the AMPs from the material surface by means of a matrix while immobilization. This type of matrix is called “container”. Compared to the first two immobilization methods, the main difference is that there is no direct physical contact between the AMPs and the material surface, and the “container” can provide more diverse release modes for the AMPs (Li et al., 2017; Liu H et al., 2018; Chen J et al., 2020). For example, Liu et al. prepared a nano-hydroxyapatite coating on the surface of a titanium plate by impregnation coating (Liu Z et al., 2018). The bifunctional titanium material was then developed by the physical properties of nano-hydroxyapatite particles to adsorb HBD-3 and BMP-2. The slow and simultaneous release of HBD-3 and BMP-2 conferred the scaffold with long-term resistance to biofilm and the ability to promote osteogenic differentiation. Adsorption of AMPs using nano-hydroxyapatite coating can avoid complex chemical processes and the effect of catalyst residues on peptide activity. In addition, titanium dioxide nanotubes are widely used in the study of surface loading of AMPs on titanium implants because of their excellent biocompatibility as carriers of biofactors and their ability to provide local slow release function (Chen X et al., 2020; Wang L et al., 2022). Wang et al. used anodic oxidation and hydrothermal synthesis to prepare uniformly distributed and well-arranged TNTs structures on the surface of titanium plates (Wang B et al., 2022). The fabricated TNTs were hollow tube-like structures, which provided the structural basis for subsequent high loading of AMPs. Then, they applied the vacuum-assisted physisorption method to load the fused peptide (HHC36-RGD) into the TNTs. Further studies demonstrated that the titanium implants constructed by Liu were effective in preventing bacterial infection and promoting early osseointegration, reconciling the conflict between traditional antimicrobial materials and osteogenic surfaces, and were a promising material for anti-infection bone repair. However, the release pattern exhibited by TNTs - “abrupt release, slow release, near linear release” - is only theoretically consistent with the conventional antimicrobial process. To this end, Chen et al. developed a titanium dioxide nanotube structure with PH-controlled release, enabling smart drug delivery on demand (Chen J et al., 2020). Specifically, after loading HHC36 inside the TNTs, they closed the outer mouth of the nanotubes with a pH-responsive molecular gate, polymethylmethacrylate (PMAA). pMAA swelled in pH ≈ 7.4 (physiological environment without bacterial infection), preventing the abrupt release of AMPs and thus mitigating cytotoxicity; in contrast, PMAA in pH ≤ 6.0 (acidic microenvironment produced by bacterial infection) disintegrates and rapidly releases AMPs showing powerful bactericidal activity (Chen X et al., 2020). A rabbit tibial infection model confirmed the biocompatibility, responsiveness and sterilization activity of this Pandora’s box-like controlled release system. This upgrade from slow release to controlled release is certainly a goal to be pursued for future drug delivery system. Moreover, it has been suggested that titanium nanopores (NPs) structures have stronger bonding strength to titanium plates compared to nanotubes (NTs) and have better biocompatibility and osteogenesis induction. Shen et al. fabricated NPs and NTs on the surface of titanium plates by varying the process parameters and loaded the antimicrobial peptide LL37 onto them, respectively (Shen et al., 2019). By comparison, they verified that the NPs coating is more stable than the NTs coating and seems to be more suitable for the preparation of AMP-loaded combination titanium implants for bone injury treatment.

#### 4.1.2 Nanoparticles

Nanoparticles are an effective tool for local delivery of drugs and have been widely used in recent years for topical application of bioactive factors. Delivery of AMPs with nanoparticles has also gradually become a research hotspot to assist in the repair of infected bone defects. Commonly used nanoparticles for drug delivery system include polymeric microspheres and inorganic particles (He et al., 2018a; He et al., 2020; Honda et al., 2020). Honda et al. noticed that the amine group of fish sperm protein arginine adsorbed with the phosphate group and hydroxyl group of HAp through electrostatic interactions, so they loaded fish sperm protein onto HAp by intermittent adsorption and compressed it into bone implants (Honda et al., 2020). It has the osteogenic properties expected of a bone scaffold and the antimicrobial activity conferred by AMPs. In addition, this team found that HAp-adsorbed fisetin is released by ion exchange, so the salt concentration in the local environment can modulate the release of AMPs from the nanoparticles (Honda et al., 2020). Another team used negatively charged filamentous protein particles (SFNPs) to adsorb positively charged LL37 and crosslink it to the surface of the titanium scaffold (He et al., 2019). LL37 was able to recruit MSCs and macrophages and induce M2 polarization, which significantly promoted bone formation around the titanium scaffold. Since positive charge is one of the characteristics of AMPs, negatively charged SFNPs are a universal platform for loading AMPs.

Relying solely on physical adsorption tends to produce high levels of synaptic release, which shows some cytotoxicity. However, encapsulation of AMPs with degradable polymeric microspheres to degrade and release it per microsphere unit can reduce the sudden release due to binding instability. He et al. prepared PLGA microspheres encapsulated with Pac-525 and KSL-W using electrospray technology, and the encapsulation rate of PLGA microspheres up to more than 90% was observed by electron microscopy, which confirmed the drug loading performance of PLGA microspheres (He et al., 2020). With the degradation of microspheres, AMPs was slowly released in small dose units, and studies showed that its long-term antimicrobial effect was very promising (>35 days). In addition, AMP-loaded PLGA microspheres mounted on porous mineralized collagen scaffolds exhibited excellent antibacterial and osteogenic activities (He et al., 2020). In addition, AMP-loaded nanoparticles can be attached to other forms of implants to accomplish drug delivery, such as metal scaffolds, fibrous membrane scaffolds, *etc.* For example, He’s team also used the aforementioned PLGA microspheres for the osteogenic layer in a layer-by-layer fibrous membrane scaffold to produce a guided bone regeneration (GBR) membrane with antimicrobial properties (He et al., 2018b). Tian et al. coated HA particles loaded with AMPs analogs on the surface of a magnesium alloy scaffold, which improved the corrosion resistance of the magnesium alloy while providing anti-biofilm properties (Tian et al., 2015). The modified magnesium alloy scaffold perfectly repaired the femoral condylar injury in rabbits. Clearly, the protection, delivery and slow release of AMPs with degradable polymeric microspheres offer a promising future for the treatment of infected bone defects.

#### 4.1.3 Hydrogels

In the field of tissue engineering, hydrogels are considered to be ideal scaffolds for delivery of active factors. In recent years, researchers have been gradually using hydrogel scaffolds to deliver AMPs for infectious tissue repair. Yang et al. successfully combined RADA16 and Tet213 to make a self-assembled hydrogel scaffold for the treatment of osteomyelitis (Yang et al., 2018). It is known that RADA16 peptide hydrogel consists of a high mass ratio of water, can self-assemble under physiological conditions, and it has the potential to induce new bone production. Yang et al. loaded AMPs into RADA16 without changing its appearance and properties. The novel hydrogels were produced with a dense nanofiber network structure, which on the one hand has antibacterial ability and on the other hand can provide a suitable growth environment for bone marrow mesenchymal stem cells and promote the proliferation of osteoblasts (Yang et al., 2018). The production process of AMPs delivery by means of hydrogels is simple and the resulting novel hydrogels can be applied by local injection. This also exposes the shortcomings of its osteoconductivity. Therefore, the use of hydrogel-loaded drugs alone for bone defect repair is currently rare. To this end, Cheng et al. made a coating of AMP-loaded methacrylate-based hydrogel (GelMA), with titanium providing the necessary mechanical properties and osteoconductivity of the bone scaffold (Cheng et al., 2017). This novel hydrogel coating consists of silicate nanoparticles to provide bone-enabling activity, antimicrobial peptide HHC36 to provide antimicrobial activity, and catechol moieties to provide adhesion. In addition, AMP is released from the hydrogel in a diffusive manner due to the absence of chemical coupling between HHC36 and the hydrogel backbone, which may lead to a rapid release of AMPs. To achieve long-term release, Cheng enhanced the binding strength of AMP by photo-crosslinking (Cheng et al., 2017). The resulting novel hydrogel coating exhibited stable antibacterial ability. In conclusion, hydrogels are good scaffolds for *in vivo* protection and delivery of AMPs, but AMPs release kinetics and its application to bone tissue engineering scaffolds need to be further investigated.

### 4.2 Gene therapy by antimicrobial peptides

In recent years, gene therapy involves strategies to transfer nucleic acids encoding target proteins or peptides to repair cells such as MSCs, thus promoting cell differentiation and tissue regeneration (Atasoy-Zeybek and Kose, 2018). And gene therapy could fulfill long-term expressions of target factors and avoid shortcomings about direct applications of protein or peptides, such as burst release and protease degradation. So transferring genes which encode AMPs to stem cells is a potential technique to treat infection diseases. Zhou et al. has transferred LL-37 gene to distal airway stem cells which were then transplanted to infected lung foci, and found that these engineered cells could express LL-37 and prevent bioengineered artificial lung from infection (Zhou et al., 2020). Therefore, using engineered stem cells which express AMPs is a promising strategy to repair infection bone defects, which needs further attention.

Depending on the vector types, gene therapy strategies can be grouped into viral gene therapy and non-viral gene therapy. And non-viral strategies are better than viral techniques on account of high biosafety and low cost. But one apparent obstacle is low level of transfection efficiency. AMPs could help to improve transfection capacity in gene therapy by enhancing cellular and nucleus entry. Peng et al. used AMP conjugated gold nanoparticles to delivery plasmid DNA (pDNA) to MSC, and found that the transfection efficiency was dramatically improved by AMP (Peng et al., 2016). And another research group verifies that an AMP, θ-defensin, can also effectively used to load and carry miRNA to MSCs, with higher efficiency than common polyethylenimine and Lipofectamine 3,000 (Yu et al., 2017). Although some AMPs potent ability to induce pDNA to stem cells, they have not been used to induce pDNA encoding osteoinductive proteins or peptides to be transferred into stem cells for tissue engineering, especially for bone regeneration. Besides, it remains unknown whether the antimicrobial properties combined with ability to improve transfection efficiency will be simultaneously maintained *in vivo*. And gene-activated matrix incorporated scaffolds, repair cells, interest genes and AMPs are also highly needed established for bone tissue engineering.

## 5 Discussion

One major challenge is the emergence of multi-resistant bacteria, so traditional antibiotics gradually fail to meet current requirements in clinic practice. And bone infection during bone repair such as the repair of fracture or infection-caused bone defects is devasting to patients and the society. Antimicrobial peptides are emerging bactericidal agents to replace traditional antibiotics because of broad spectrum antimicrobial activities and limited resistance. And there are many kinds ofAMPs, which be categorized to helix-based AMPs, sheet-based AMPs, coil-based AMPs and composite AMPs depending on their distinctive structures. These AMPs show great promise for the prevention and treatment of bone infections during bone repair, as they can exert plenty of effects which include bactericidal activity, anti-biofilm properties, immunomodulation. And AMPs could also promote bone regeneration by immunomodulation to regulate immune-microenvironment. Besides, AMPs could promote angiogenesis and modulate MSC behaviors. Regarding the application of AMPs, not only can they be immobilized on the surface of bone implant biomaterials, but also they can be incorporated into biomaterials, and related controlled release techniques have been developed. Furthermore, AMPs can be used in gene therapy to help nucleic acid delivery.

Although the application of antimicrobial peptides *in situ* for bone tissue engineering has great prospects, the number of antimicrobial peptides is so huge that it is highly needed to screen. And some parent AMPs also need be modified to design related derivatives. The overall purposes of screening and modification are to improve antibiological activity, reduce cytotoxicity and hemolysis, and enhance enzyme resistance. Besides, concise mechanisms about modes of action of AMPs remain unclear, especially anti-biofilm and immunomodulate mechanisms. In addition, AMPs are facing many challenges in bone tissue engineering applications. First, how to ensure the activity of AMPs during the delivery process. Simplifying the loading process and reducing the use of chemical reagents is the key to solving this problem. Although there are many strategies for delivering AMPs in bone tissue engineering, most of them are cumbersome in terms of steps, which not only consume human and material resources, but also are very detrimental to ensure the activity of AMPs. Therefore, there is an urgent need to develop simple and effective load strategies. Secondly, how to avoid the initial burst release of AMPs as well as to ensure a suitable local long-term peptide concentration. Excessive concentrations can lead to cytotoxicity, while insufficient concentrations make it difficult to achieve the desired effect, so a balance between cytotoxicity and effectiveness needs to be obtained. Increasing the drug loading and imparting slow-release properties or even controlled release properties seems to be the key factors to solve this problem. Although some teams have explored AMPs-related controlled release materials, there are few relevant studies in the field of bone tissue engineering. Breakthroughs in smart release studies could lead to better utilization of AMPs for bone tissue engineering in the future. Finally, how to ensure the osteogenesis-related properties of the material itself while providing biofilm resistance. This is an area that current researchers are working to address, but not doing enough. The dominant approach currently is to synthesize multifunctional chimeric peptides using solid-phase synthesis techniques, where peptide sequences with osteogenic activity are chimerized with AMPs. The limitations of this scheme are the high cost and the difficulty to ensure the function of multiple peptide sequences simultaneously. In fact, there are some AMPs, such as LL37, which themselves have osteogenic, immunomodulatory or angiogenic properties. Further exploration and discovery of more AMPs with osteogenic activity or the use of their osteogenic mechanisms to synthesize more effective AMPs could solve this problem. Therefore, researchers are needed in the future to explore the molecular mechanisms of osteogenesis of AMPs in greater depth and lay the foundation for the application of AMPs in bone tissue engineering. In conclusion, AMPs with limited resistance show great promise to be used as alternatives to traditional antibiotics for bone defects susceptible to infections, which may open a brilliant prospect for bone tissue engineering to treat clinic bone defects.
